# Prevalence of Diabetes and Impaired Fasting Glucose in Hypertensive Adults in Rural China: Far from Leveling-Off

**DOI:** 10.3390/ijerph121114764

**Published:** 2015-11-19

**Authors:** Shasha Yu, Zhaoqing Sun, Liqiang Zheng, Xiaofan Guo, Hongmei Yang, Yingxian Sun

**Affiliations:** 1 Department of Cardiology, The First Hospital of China Medical University, 155 Nanjing North Street, Heping District, Shenyang 110001, China; E-Mails: yidasasa047717@126.com (S.Y.); guoxiaofan1990@126.com (X.G.); yanghongmeidy@126.com (H.Y.); 2 Department of Cardiology, Shenjing Hospital of China Medical University, Shenyang 110001, China; E-Mail: sunzhaoqing047717@126.com; 3 Department of Clinical Epidemiology, Shenjing Hospital of China Medical University, Shenyang 110001, China; E-Mail: zhengliqiang047717@126.com

**Keywords:** diabetes, impaired fasting glucose, prevalence, related factor, hypertension

## Abstract

In recent years data from many investigations has shown a leveling–off trend in diabetes incidence. In order to explain the diabetes epidemic in rural China during the past ten years, we conducted a survey from July 2012 to August 2013. Data from comprehensive questionnaires, physical examinations, and blood tests were obtained from 5919 residents with hypertension, aged ≥ 35 years. Diabetes and impaired fasting glucose (IFG) were defined according to the American Diabetes Association (ADA) criteria. The overall prevalence of diabetes and IFG were 15.3% (13.6% in men, 16.8% in women) and 40.7% (44.1% in men, 34.7% in women) in the hypertensive rural Chinese population. The prevalence of previously diagnosed diabetes was 6.5% (4.6% in men, 8.4% in women). The prevalence of undiagnosed diabetes was 8.7% (9.0% in men, 8.5% in women). Multivariate logistic regression revealed that increasing age, drinking, overweight or obesity, systolic blood pressure, low HDL-C, high total cholesterol and triglycerides increased the risk of diabetes (*p* < 0.05). Diabetes is thus still prevalent in rural areas of China and is manifesting an accelerating trend. It remains an important public health problem in China, especially in rural areas and routine assessment for the early detection and treatment of diabetes should be emphasized.

## 1. Introduction

Diabetes is one of the most common non-communicable diseases (NCDs). It is the fourth or fifth leading cause of death in most high-income countries and there is substantial evidence that it is epidemic in many economically developing and newly industrialized countries [1]. In most countries diabetes has increased alongside rapid cultural and social changes—an aging population, the obesity epidemic, increasing urbanization, dietary changes, reduced physical activity and unhealthy behaviors [2]. Furthermore, substantial reductions in mortality related to improvement in survival from cardiovascular diseases have also promoted the growth in the prevalence of diabetes [3].

However, recent epidemic data suggests that the prevalence of prediabetes or overt diabetes may have leveled-off. Abraham and colleagues further analyzed the Framingham Heart Study data and reported that the overall annualized rates of diabetes per 1000 individuals were 3.0 in the 1970s, 4.1 in the 1980s, 6.0 in the 1990s, and 5.5 in the 2000s [4]. They concluded that incidence of diabetes had not continued to increase in the past few years. Besides, the current National Health Interview Survey (NHIS) also reported that the annual percentage change in diabetes incidence might have declined from 2008–2012 compared with 1990–2008 [5]. Other than that, a leveling-off of diabetes incidence in recent years has been seen in New York, the United Kingdom, and Denmark [6,7,8]. 

Nevertheless, most of the studies supporting this argument were conducted in developed or urban areas with a highly developed social economy and health system. In China, the economy of urban areas has undergone rapid development during the past few decades, but 57% of Chinese residents live in rural areas with relatively lower economic and medical attention levels. Besides, the new rural cooperative medical care system has just been established in rural areas. Our recent investigation showed that cardiovascular diseases like hypertension, metabolic syndrome, dyslipidemia and obesity was still showing an acceleration trend in rural China [9,10,11,12]. Furthermore, our previous study conducted during 2004–2006 confirmed that the prevalence of diabetes and IFG (10.0% and 26.0%) was higher in the hypertensive population than in the general population [13].

During the past ten years, the prevalence of hypertension in rural Northeast China increased from 36.2% to 51.1% [14]. However, there is a lack of recent data on the prevalence and epidemiologic characters of diabetes and IFG among rural residents, especially those with hypertension. The objective of the current study was to update the prevalence of diabetes and IFG and their associated factors among the hypertensive rural Chinese population.

## 2. Methods

### 2.1. Study Population

Liaoning Province is located in Northeast China. Our previous study conducted during 2004–2006 was based on a large-scale epidemiological study conducted in China with a cross-sectional survey that adopted a multi-stage, stratified clustering sampling scheme in the rural areas of Fuxin County, Liaoning Province, China. Subjects who were over 35 years of age were examined. Information on demographic characteristics including age, gender, education, ethnicity, occupation, and household income was collected. From January 2012 to August 2013, a representative sample of participants aged ≥35 years was selected to characterize the prevalence, incidence and natural history of cardiovascular risk factors in rural areas of Liaoning Province. The study adopted a multi-stage, stratified, random-cluster sampling scheme. In the first stage, three counties (Dawa, Zhangwu and Liaoyang County) were selected from the eastern, southern and northern regions of Liaoning Province. In the second stage, one town was randomly selected from each county (for a total of three towns). In the third stage, 8–10 rural villages from each town were randomly selected (for a total of 26 rural villages). Participants who were pregnant or had malignant tumors or mental disorders were excluded from the study. All the eligible permanent residents aged ≥35 years from each village were invited to attend the study (a total of 14,016 participants). Of those, 11,956 participants agreed and completed the study to give a response rate of 85.3%. The study was approved by the Ethics Committee of China Medical University (Shenyang, China). All procedures were performed in accordance with ethical standards. Written consent was obtained from all participants after they had been informed of the objectives, benefits, medical items and confidentiality agreement regarding their personal information. For participants who were illiterate, we obtained written informed consent from their proxies. In this report, we used only the data from participants who completed the study, which provided a final sample size of 5919 (2892 men and 3027 women).

### 2.2. Data Collection and Measurements

Data were collected during a single visit to the clinic by cardiologists and trained nurses using a standard questionnaire in a face-to-face interview. Before the survey was performed, we invited all eligible investigators to attend an organized training session. The training included the purpose of this study, how to administer the questionnaire, the standard method of measurement, the importance of standardization and the study procedures. A strict test was administered after this training, and only those who scored perfectly on the test were accepted as investigators in this study. During data collection, our inspectors had further instructions and support.

Data regarding the demographic characteristics, lifestyle risk factors, dietary habits, family income and family history of chronic diseases were obtained during the interview using the standardized questionnaire. The study was guided by a central steering committee with a subcommittee for quality control. Educational level was assessed as completion of primary school or less, middle school or high school and higher. Self-reported sleep duration (including nocturnal and nap duration) was obtained from the questionnaire. Family income was classified as ≤5000 CNY/year (788 dollar/year), 5000–20,000 (788–3152 dollar/year) and >20,000 CNY/year (3152 dollar/year).

According to the American Heart Association protocol, blood pressure (BP) was measured three times at 2-min intervals after at least 5 min of rest using a standardized automatic electronic sphygmomanometer (HEM-907; Omron Healthcare, Kyoto, Japan), which had been validated according to the British Hypertension Society protocol [15]. The participants were advised to avoid caffeinated beverages and exercise for at least 30 min before the measurement. During the measurement, the participants were seated with their arms supported at the level of the heart. The mean of three BP measurements was calculated and used in all analyses.

Weight and height were measured to the nearest 0.1 kg and 0.1 cm, respectively, with the participants wearing light-weight clothing and without shoes. Waist circumference (WC) was measured at the umbilicus using a non-elastic tape (to the nearest 0.1 cm), with the participants standing at the end of normal expiration. Body mass index (BMI) was calculated as the weight in kilograms divided by the square root of the height in meters.

Fasting blood samples were collected in the morning after at least 12 h of fasting. Blood samples were obtained from an antecubital vein into Vacutainer tubes containing ethylenediaminetetraacetic acid (EDTA). Fasting plasma glucose (FPG), total cholesterol (TC), low-density lipoprotein cholesterol (LDL-C), high-density lipoprotein cholesterol (HDL-C), triglycerides (TGs) and other routine blood biochemical indexes were analyzed enzymatically using an autoanalyzer. All laboratory equipment was calibrated, and blinded duplicate samples were used for these analyses.

### 2.3. Definitions

Diabetes was defined as a fasting plasma glucose (FPG) value ≥7.0 mmol/L or a previous diagnosis of diabetes. Diabetes was further subclassified as “known diabetes” if diabetes had been diagnosed previously by a medical practitioner or as “newly diagnosed diabetes” if diabetes was first diagnosed by this study [16]. A self-reported history of diabetes was confirmed by the use of insulin or oral hypoglycaemic agents. Two definitions for IFG were used in this study. By the 1997 ADA criteria, IFG was defined as fasting plasma glucose value of 6.1–6.9 mmol/L in the absence of a previous diagnosis of diabetes [17]; by the 2014 ADA criteria, IFG ws defined as a fasting plasma glucose value of 5.6–6.9 mmol/L in the absence of a previous diagnosis of diabetes [18].

Hypertension was considered present if any of the following conditions were met: systolic blood pressure ≥140 mmHg, diastolic blood pressure ≥90 mmHg, or reported use of a medication for hypertension and the classification of BP in response to JNC8 [19]. According to the World Health Organization criteria, BMI was categorized into two groups as normal (BMI < 25), overweight (30 > BMI ≥ 25) and obesity (BMI ≥ 30) [20].

For most age-related comparisons, participants were separated into four groups according to age (35–44 years, 45–54 years, 55–64 years, ≥65 years). Physical activity included occupational and leisure-time physical activity. A detailed description of the methods for assessing physical activity has been presented elsewhere [21]. Occupational and leisure-time physical activity were merged and regrouped into the following three categories: (1) low—subjects who reported light levels of both occupational and leisure-time physical activity, (2) moderate—subjects who reported moderate or high levels of either occupational or leisure-time physical activity and (3) high—subjects who reported a moderate or high level of both occupational and leisure-time physical activity. Dietary patterns were assessed by having participants recall the foods they had eaten during the previous year. According to the Dietary Guidelines for Chinese Residents, a dietary pyramid has been developed to describe the Chinese dietary pattern (*i.e.*, daily consumption of cereals and products, vegetables, fruits, weekly consumption of fish, poultry, egg; milk and production, soybean and products; oil and salt). Based on this dietary pattern and the reported monthly frequency consumption of various food groups, we calculated a special diet score for each participant that assessed adherence to the Chinese diet (range 0 to 6). The questionnaire included questions regarding the average consumption of several food items per week. The reported consumption was quantified approximately in terms of grams per week. Those questions include vegetable consumption and meat consumption. Vegetable consumption was assessed on the following scale: rarely = 3, <1000 g = 2, 1000–2000 g = 1, ≥2000 g = 0, and meat consumption, including red meat, fish and poultry was assessed on the following scale: rarely = 0, <250 g = 1, 250–500 g = 2 and ≥500 g = 3). Higher values of the diet score indicated higher meat consumption, lower vegetable consumption and greater adherence to a Westernized diet, while lower values indicate adherence to the Chinese diet. Similar methods for calculating a diet score can be found in the ATTICA study [22].

### 2.4. Statistical Analysis

Descriptive statistics were calculated for all the variables, including continuous variables (reported as mean values and standard deviations) and categorical variables (reported as numbers and percentages). The differences between different groups were evaluated using the Student’s *t*-test, ANOVA, non-parametric test or the χ^2^-test, as appropriate. Multinomial logistic regression analyses were used to identify independent factors of IFG and Diabetes, and odds ratios (ORs) and corresponding 95% confidence intervals (CIs) were calculated. All the statistical analyses were performed using SPSS version 17.0 software (SPSS Inc., Chicago, IL, USA), and *p* values less than 0.05 were considered statistically significant.

## 3. Results

Characteristics of the hypertensive residents enrolled in this study, as stratified by gender, are shown in Table 1. All subjects were between 35 and 92 years old and the average ages of the male and female residents were 57.23 ± 10.63 and 57.35 ± 9.87 years, respectively. Compared with men, hypertensive women had higher levels of BMI, total cholesterol, LDL-C and higher proportion of Light physical activity (*p* < 0.001), but levels of diet score, sleep duration and diastolic blood pressure, smoking and drinking status in men than in women (*p* < 0.001). Characteristics of study population with or without diabetes and IFG were shown in Table 2. Those with abnormal glucose metabolism were more likely to be Han and had lower rate of both severe and light physical activity. They were less likely to be current smoker. Those IFG and diabetic residents had higher mean values of BMI, SBP, DBP, FPG, TG, LDL-C and total cholesterol and relatively lower values of HDL-C.

**Figure 1:**
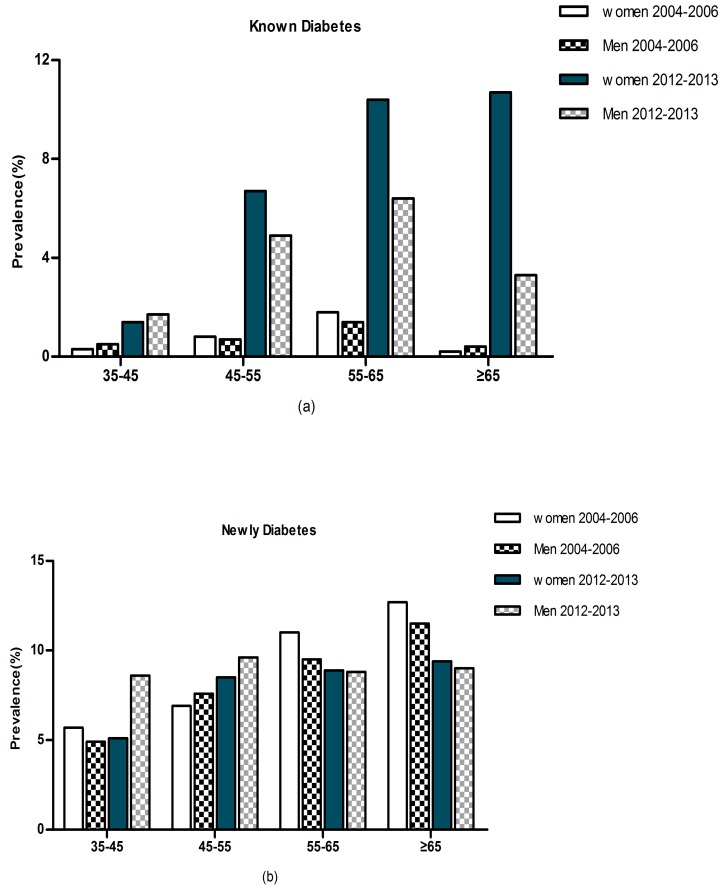
Prevalence of (**a**) known diabetes, (**b**) newly diagnosed diabetes, by gender and age from 2004–2006 years to 2012–2013 years in hypertensive subjects in rural Northeast China.

**Figure 2:**
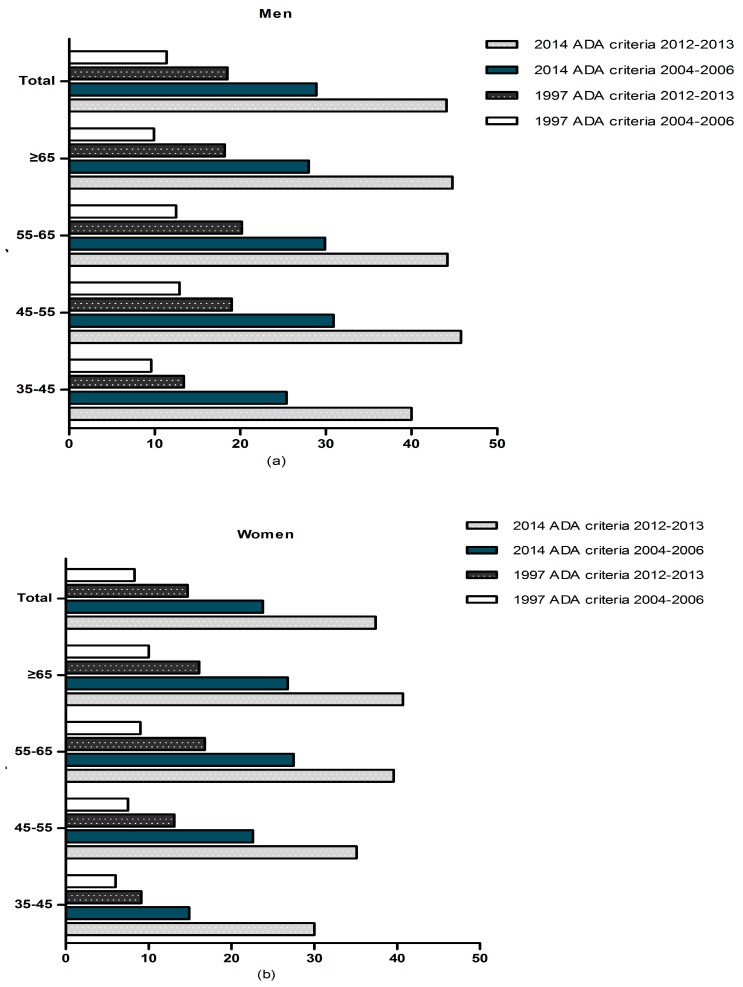
Prevalence of IFG from 2004–2006 years to 2012–2013 years by age in hypertensive (**a**) men, (**b**) women, in rural Northeast China.

Figure 1 and Figure 2 show the prevalence of diabetes and IFG by age and gender from 2004–2006 years to 2012–2013 years. Overall the prevalence of diabetes was 15.3% (13.6% in men and 16.8% in women) in our present study. The prevalence of self-reported diabetes in women was higher than men (8.4% and 4.6%, respectively). We found that 8.7% subjects had previously undiagnosed diabetes (9.0% in men and 8.5% in women). In addition, the prevalence of IFG was 40.7% (44.1% in men and 37.4% in women, *p* < 0.05) in the study population according to the 2014 ADA criteria. In contrast, when the previous IFG criterion (fasting plasma glucose 6.1–6.9 mmol/L) was used, the prevalence of IFG fell to 16.5% (18.5% in men and 14.7% in women, *p* < 0.05). From 2004–2006 years to 2012–2013 years, there was a significant increasing of prevalence of diabetes and IFG in rural Northeast China (13.6% *vs.* 9.7% in men and 16.8% *vs.* 10.2% in women). Compared with 2004–2006, the data from 2012–2013 showed that the prevalence of known diabetes had increased dramatically. Prevalence of newly diagnosed diabetes increased with age in women and reached its peak in the oldest female age group. Besides, the prevalence of self-reported diabetes also show the same trend. However, in men this situation is not exist. In men, prevalence of self-reported diabetes increased with age and reached its peak in 55–65 years of age, and after that, it decreased significantly in those ≥65 years. On the other sides, prevalence of newly diagnosed diabetes increased with age in men but reached its peak in 45–55 years. 

**Table 1:** Demographic and anthropometrical characteristics of the study population.

Variables	Men (*n* = 2892)	Women (*n* = 3027)	*p* values
Age (year)	57.23 ± 10.63	57.35 ± 9.87	0.647
BMI (kg/m^2^)	25.37 ± 3.53	25.75 ± 3.77	<0.001
Diet score	2.53 ± 1.12	2.08 ± 1.10	<0.001
Sleep duration (h/d)	7.45 ± 1.71	7.03 ± 1.79	<0.001
Mean systolic blood pressure (mmHg)	159.06 ± 19.04	158.66 ± 20.23	0.433
Mean systolic blood pressure (mmHg) ^**#**^	158.18 ± 17.42	157.01 ± 16.50	0.029
Mean diastolic blood pressure (mmHg)	90.26 ± 11.17	87.46 ± 11.00	<0.001
Mean diastolic blood pressure (mmHg) ^**#**^	89.75 ± 10.60	86.68 ± 10.17	<0.001
Fasting plasma glucose (mmol/L)	6.14 ± 1.86	6.18 ± 1.92	0.413
Fasting plasma glucose (mmol/L) ^**##**^	5.98 ± 1.49	5.94 ± 1.55	0.307
Total cholesterol (mmol/L)	5.31 ± 1.05	5.52 ± 1.16	<0.001
Triglycerides (mmol/L)	1.80 ± 1.76	1.86 ± 1.55	0.122
LDL-C (mmol/L)	2.99 ± 0.82	3.16 ± 0.88	<0.001
HDL-C (mmol/L)	1.43 ± 0.45	1.41 ± 0.36	0.240
Current smoking status, %	1551 (53.6)	525 (17.3)	<0.001
Current drinking status, %	1352 (46.7)	98 (3.2)	<0.001
Ethnicity			0.347
Han	2742 (94.8)	2862 (94.5)	
Others *	150 (5.2)	165 (5.5)	
Educational status			<0.001
Primary school or below	1303 (45.1)	1999 (66.0)	
Middle school	1249 (43.2)	848 (28.0)	
High school or above	340 (11.8)	180 (5.9)	
Physical activity			<0.001
Light	783 (27.1)	1229 (40.6)	
Moderate	1935 (66.9)	1626 (53.7)	
Severe	174 (6.0)	172 (5.7)	
Annual income (CNY/year)			0.006
≤5000	439 (15.2)	468 (15.5)	
5000–20,000	1569 (54.3)	1746 (57.7)	
>20,000	884 (30.6)	813 (26.9)	

Data are expressed as the mean ± SD or as *n* (%). Abbreviations: CNY, China Yuan (1 CNY = 0.161 USD); * Including some ethnic minorities in China, such as Mongol and Manchu; ^**#**^ exclude those already in hypertension medication; ^**##**^ exclude those those already in diabetes medication.

**Table 2:** Characteristics of study population with or without diabetes and IFG in the rural hypertensive population of Liaoning Province, China.

Variables	Normal (*n* = 2607)	IFG (*n* = 2409)	Diabetes (*n* = 903)	*p* values
Age (year)	56.43 ± 10.76	57.74 ± 10.00	58.58 ± 9.12	<0.001
Ethnicity				<0.001
Han	2434 (93.4)	2313 (96.0)	857 (94.9)	
Others *	173 (6.6)	96 (4.0)	46 (5.1)	
Educational status				0.367
Primary school or below	1433 (55.0)	1341 (55.7)	528 (58.5)	
Middle school	936 (35.9)	865 (35.9)	296 (32.8)	
High school or above	238 (9.1)	203 (8.4)	79 (8.7)	
Physical activity				<0.001
Light	841 (32.3)	799 (33.2)	372 (41.2)	
Moderate	1630 (62.5)	1460 (60.6)	471 (52.2)	
Severe	136 (5.2)	150 (6.2)	60 (6.6)	
Annual income (dollar/year)				0.187
≤788	417 (16.0)	362 (15.0)	128 (14.2)	
788–3152	1481 (56.8)	1337 (55.5)	497 (55.0)	
>3152	709 (27.2)	710 (29.5)	278 (30.8)	
Current smoking (yes)	962 (36.9)	854 (35.5)	260 (28.8)	<0.001
Current drinking (yes)	583 (22.4)	671 (27.9)	196 (21.7)	<0.001
Diet score	2.30 ± 1.13	2.33 ± 1.13	2.18 ± 1.12	0.003
Sleep duration (h/d)	7.31 ± 1.73	7.18 ± 1.72	7.18 ± 1.95	0.019
BMI (kg/m^2^)	25.22 ± 3.71	25.57 ± 3.54	26.53 ± 3.66	<0.001
SBP (mmHg)	156.94 ± 16.62	159.42 ± 17.80	159.16 ± 17.77	<0.001
DBP (mmHg)	88.14 ± 10.43	89.24 ± 10.55	88.02 ± 10.87	0.04
FPG (mmol/L)	5.40 ± 0.42	6.43 ± 0.24	9.17 ± 2.93	<0.001
Total cholesterol (mmol/L)	5.26 ± 1.05	5.49 ± 1.09	5.68 ± 1.26	<0.001
Triglycerides (mmol/L)	1.58 ± 1.16	1.82 ± 1.46	2.59 ± 2.76	<0.001
LDL-C (mmol/L)	2.99 ± 0.83	3.12 ± 0.85	3.22 ± 0.94	<0.001
HDL-C (mmol/L)	1.46 ± 0.42	1.41 ± 0.40	1.32 ± 0.35	<0.001

Data are expressed as the mean ± SD or as *n* (%); Abbreviations: SBP, systolic blood pressure; DBP, diastolic blood pressure; FPG, fasting plasma glucose; CNY, China Yuan (1 CNY = 0.161 USD); * Including some ethnic minorities in China, such as Mongol and Manchu.

**Figure 3:**
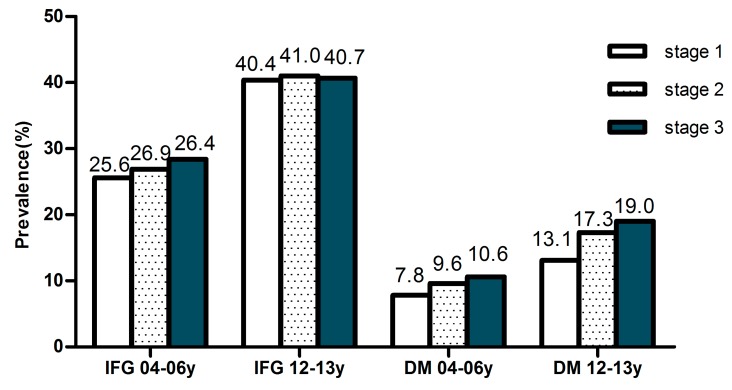
Prevalence of diabetes and IFG by classification of blood pressure. 04–06y means from 2004–2006 years, 12–13y means form 2012–2013 years.

The prevalence of IFG also increased with age among men and women according to the 2014 ADA criteria, although there was a slight decline among men and women above the age of 65 years. The prevalence of diabetes and IFG also increased with the increased stage of blood pressure (shown in Figure 3). By using multivariate logistic regression analysis which shown in Table 3, increasing age, current drinking, overweight or obesity, systolic blood pressure, high total cholesterol, high triglycerides and low HDL-C were determined to be related factors for diabetes (*p* < 0.05). Race (Han), increasing age, sex (male), current drinking, overweight or obesity, diastolic blood pressure, high total cholesterol and low HDL-C were determined to be related factors for IFG (*p* < 0.05).

**Table 3:** Risk factors for diabetes and IFG determined by multinomial logistic regression analysis.

Variables	Diabetes *		IFG *	
	OR (95% CI)	*p* values	OR (95% CI)	*p* values
Gender (Male)	1.008 (0.825, 1.232)	0.937	1.207 (1.046, 1.392)	0.010
Race (Han)	1.297 (0.913, 1.843)	0.147	1.609 (1.239, 2.088)	<0.001
Age (per one year)	1.022 (1.012, 1.033)	<0.001	1.019 (1.012, 1.026)	<0.001
Educational status				
High school or above	1.000 (reference)		1.000 (reference)	
Middle school	1.035 (0.768, 1.394)	0.822	1.147 (0.926, 1.421)	0.209
Primary school or below	0.963 (0.715, 1.296)	0.802	1.097 (0.885, 1.359)	0.400
Physical activity				
Severe	1.000 (reference)		1.000 (reference)	
Moderate	0.796 (0.571, 1.110)	0.179	0.837 (0.647, 1.083)	0.176
Light	0.950 (0.675, 1.337)	0.768	0.857 (0.669, 1.098)	0.222
Annual income, dollar/year				
≤788	1.000 (reference)		1.000 (reference)	
788–3152	0.810 (0.675, 0.973)	0.024	0.892 (0.781, 1.019)	0.092
>3152	0.662 (0.509, 0.863)	0.002	0.823 (0.681, 0.994)	0.044
Current smoking	0.705 (0.583, 0.851)	<0.001	0.814 (0.713, 0.929)	0.002
Current drinking	1.337 (1.059, 1.688)	0.015	1.381 (1.177, 1.620)	<0.001
Body mass index, kg/m^2^				
<25	1.000 (reference)		1.000 (reference)	
25–30	1.419 (1.190, 1.693)	<0.00	1.162 (1.027, 1.313)	0.017
≥30	2.121 (1.638, 2.746)	<0.001	1.288 (1.048, 1.582)	0.016
Diet score (per one point)	0.929 (0.864, 0.999)	0.048	1.016 (0.964, 1.070)	0.553
Sleep duration (per one hour)	0.962 (0.920, 1.007)	0.095	0.952 (0.922, 0.984)	0.003
SBP (per one mmHg)	1.008 (1.003, 1.013)	0.001	0.998 (0.994, 1.001)	0.998
DBP (per one mmHg)	0.994 (0.986, 1.003)	0.204	1.009 (1.002,1.015)	0.009
Total cholesterol, mmol/L				
<6.21	1.000 (reference)		1.000 (reference)	
≥6.21	1.603 (1.263, 2.035)	<0.001	1.457 (1.213, 1.749)	<0.001
Triglycerides, mmol/L				
<2.26	1.000 (reference)		1.000 (reference)	
≥2.26	2.633 (2.190, 3.165)	<0.001	1.147 (0.985, 1.336)	0.077
LDL-C, mmol/L				
<4.16	1.000 (reference)		1.000 (reference)	
≥4.16	1.101 (0.809, 1.498)	0.542	1.244 (0.976, 1.586)	0.078
HDL-C, mmol/L				
≥1.03	1.000 (reference)		1.000 (reference)	
<1.03	1.409 (1.117, 1.778)	0.004	1.312 (1.095, 1.571)	0.003

* Adjusted for gender, age, education, smoking status, drinking status, BMI, SBP, DBP, total cholesterol, triglycerides, HDL-C, and LDL-C.

## 4. Discussion

Our study indicated that elevated blood glucose (diabetes and IFG) was very common among hypertensive residents in rural Northeast China: 56% had diabetes or IFG (57.7% in men and 54.2% in women) and 15.3% had diabetes, which included 6.5% previously diagnosed and 8.7% newly diagnosed cases. Adjusting for possible confounders, increasing age, current drinking, overweight or obesity, systolic blood pressure, high total cholesterol, high triglycerides and low HDL-C increased the risk of diabetes while lower annual income, current smoking and higher diet score were correlated with lower rates of diabetes.

Most recent estimates of IDF levels indicate that 8.3% of adults—382 million people—have diabetes with 80% of the total number affected living in low- and middle- income countries [23]. In the recent few decades, prevalence of diabetes in China has increased rapidly, from less than 1% in the 1980s, to 2.5% in 1994, 2.7% in 2002 and 9.7% in 2007 [24,25,26,27]. The most recent nationwide survey in China reported that the overall prevalence of diabetes and pre-diabetes was estimated to be 11.6% and 50.1%, while the prevalence of previously diagnosed and undiagnosed diabetes was 3.5% and 8.1% [28]. Our present study, conducted in a rural area and with enrolled hypertensive residents, showed that the overall prevalence of diabetes and IFG was 15.3% and 40.7% and the prevalence of diagnosed and undiagnosed diabetes were 6.5% and 8.7%, respectively. In the meantime, we reported that there was an increasing prevalent trend of diabetes among hypertensive rural Chinese from 2004–2006 to 2012–2013 [13]. These figures were higher than a previous study of hypertensive Chinese conducted from 2008 to 2009 in Eastern China (diabetes: 13.2%; IFG: 14.1%) [29]. Unhealthy diet and sedentary life style with resultant of high prevalence of obesity might partially explain the increasing trend of diabetes in rural China [30]. It was worth mentioning that in rural China, the observed increase in the prevalence of diabetes could also be due to the use of hydrochlorothiazide as the most common antihypertensive drug which had been recognized to influence diabetes levels [31,32]. Diabetes and hypertension are well connected diseases, and it quite likely that both of these diseases present in the same patient. When this occurs they are called “comorbidities” [33]. Previous studies have confirmed that subjects with hypertension, compared with the general subjects, have higher prevalence of diabetes. Nayak and colleagues showed that as severity of hypertension increased, the prevalence of diabetes also increased (37.2% in normal BP, 42.7% in prehypertension, 61.4% in stage 1 hypertension and 63.5% at stage 2 hypertension) [34]. Followed by the increasing trend of diabetes and numbers of cases, the awareness and treatment rates stayed at a lower level. IDF has estimated that there are over 382 million diabetics, yet, 175 million cases were currently undiagnosed and 84% of the total number were living in low- and middle- income countries [23]. In rural China the diabetic awareness status also faced the same problem. In our study, newly diagnosed diabetes accounted for more than 50% of all diabetes. Encouragingly, however, from 2004 to 2012 years, the rate of diabetes awareness had significantly increased, even though it was relatively lower in the 35–45 year old group. This meant rural residents in China are beginning to become aware of and pay attention to diabetes.

Cardiovascular disease risk factors like hypertension, dyslipidemia and obesity were more common in subjects with diabetes than those without it [35]. In the multinomial logistic regression analysis, high BMI, current drinking, high systolic blood pressure and dyslipidemia significantly increased risk of diabetes after adjusting for gender, age, education, smoking status. Surprisingly, smoking was correlated with a lower prevalence of diabetes and IFG in hypertensive residents from rural Northeast China. These results are inconsistent with the results of previous studies [36,37]. With the development of a healthy education program in rural areas over the past ten years, individuals have begun to pay attention to their health. Once participants knew they had hypertension or diabetes, they made efforts to rectify their metabolic disorders by changing their living habits, like quitting smoking. This change may result in a lower prevalence of smoking in individuals with diabetes and IFG. Furthermore, Stein and colleagues found that quitting smoking was contrarily associated with increased diabetes and IFG risk [38]. All the above related factors are modifiable; hence, early screening and intervention of these related factors should be recommended and necessary preventive strategies also should be implemented.

This study was performed in a representative sample of rural Chinese residents. Liaoning Province has an average population density of 297 people per square kilometer, wherein the majority of the population is settled in rural areas; thus, the findings were likely to be generally applicable to the rural Chinese population. However, some limitations of this study must be considered. First, our study was a cross-sectional study, which restricted the interpretation of the observed associations in terms of cause and effect. Longitudinal studies are required for further investigation of these findings. Second, the prevalence of diabetes and IFG was based on a single assessment of blood samples and we did not test for glycocylated Hb (HbA1c), which may introduce errors. No oral glucose tolerance tests and test of glycosylated hemoglobin were conducted, which could have resulted in overestimation of diabetes prevalence. In addition, although the researchers had been trained according to a standardized protocol of measurements, measurements at a single visit might lead to incorrect values for the anthropometric indexes.

## 5. Conclusions

In conclusion, even if overall diabetes incidence in other developed areas appeared to be leveling off, the diabetes epidemic seems far from over, especially in rural China. In our study, over 50% of hypertensive residents in rural Northeast China had diabetes or IFG. The obvious accelerating trend of diabetes and IFG during the past ten years deserves our attention. Effective diabetic screening is in-need in hypertensive individuals and should be one of the focuses of public health in rural China.
